# Diastolic function and its association with diabetes, hypertension and age in an outpatient population with normal stress echocardiography findings

**DOI:** 10.1186/s12947-020-00228-9

**Published:** 2020-11-20

**Authors:** Martin G. Sundqvist, Anders Sahlén, Zee Pin Ding, Martin Ugander

**Affiliations:** 1 Department of Clinical Science and Education, Södersjukhuset, Karolinska Institutet, and Department of Cardiology, Södersjukhuset, SE-118 83 Stockholm, Sweden; 2 National Heart Centre, Singapore, 5 Hospital Drive, Singapore, 169609 Singapore; 3 Department of Clinical Physiology, Karolinska University Hospital, and Karolinska Institutet, Stockholm, Sweden; 4 Kolling Institute, Royal North Shore Hospital, and Northern Clinical School, Sydney Medical School, University of Sydney, Sydney, Australia

**Keywords:** Echocardiography, Diastolic function, Hypertension, Diabetes mellitus, Ageing

## Abstract

**Background:**

Diastolic dysfunction can be caused by hypertension or diabetes mellitus, and it is also often found with increasing age. In a given patient, the cause of diastolic dysfunction is therefore not always obvious. We sought to study the interplay of these risk factors for diastolic dysfunction in an outpatient population with a low likelihood of ischemic heart disease.

**Methods:**

Consecutive patients referred for stress echocardiography were included retrospectively. Exclusion criteria included pathological stress response, atrial arrhythmia, left ventricular ejection fraction < 55%, and more than mild valvular disease. Standard diastolic parameters were recorded in all patients. In a subset of patients, mechanistic analysis of early filling was performed using the parameterized diastolic filling (PDF) method.

**Results:**

We included 726 patients (median [interquartile range] age 56 (44–65) years, 57% male). The prevalence of diabetes and hypertension was 43 and 49%, respectively. In multiple linear regression modeling, the presence of diabetes, hypertension, sex and increasing age explained a moderate amount of the variance in e’ velocities, E/A ratio and E/e’ (R^2^ = 0.31–0.48, *p* < 0.001), and a low amount of the variance in left atrial volume index (LAVI) and the PDF parameters (*n* = 446, R^2^ = 0.05–0.17, *p* < 0.001). Sex was only related to LAVI and E/e’ for the conventional parameters (beta − 0.94, *p* = 0.04, and beta − 0.91, *p* < 0.001, respectively).

**Conclusions:**

Diabetes, hypertension, increasing age, and to a lesser extent sex, explain a moderate amount of the variance in conventional diastolic parameters related to myocardial tissue velocities and E/A ratio in a healthy outpatient population. The effect of these risk factors was substantially less pronounced on left atrial volume index and the PDF parameters.

## Background

The parameters used to assess diastolic function can be affected by fluctuations in normal physiology and individual differences, such as sex, as well as various pathological conditions. It is established that diastolic function deteriorates with increasing age [1], and that hypertension [2] and diabetes [3] often are associated with diastolic dysfunction. Furthermore, the increasingly recognized entity of heart failure with preserved ejection fraction (HFpEF) is commonly understood to be, at least partially, characterized by diastolic dysfunction [4]. It is therefore important to elucidate how different risk factors and patient characteristics can be expected to affect the parameters used to assess diastolic function. However, the relative importance of these risk factors is less well established. In this study, we aimed to investigate how age, sex, and the presence of hypertension and diabetes affect diastolic parameters in an outpatient setting.

The most common parameters used to assess diastolic function are the ratio between early and late transmitral velocity (E/A ratio), the early diastolic mitral annular displacement velocity (e’), and the ratio of E/e’. Furthermore, left atrial enlargement and signs of pulmonary hypertension are used as supportive signs of increased left heart filling pressure [1]. The parameterized diastolic filling formalism (PDF) method offers an alternative approach to analyzing diastolic function, using a model from mechanics to describe left ventricular recoil and filling during early diastole as represented by the E-wave as recorded by pulsed wave (PW) Doppler. Briefly, early diastolic filling is modeled as a form of damped harmonic motion, similarly to how the motion of a compressed spring upon its release would be modeled. Taking the analogy of a spring, motion in this model is described using three parameters: *x* being the distance from the equilibrium point (e.g. distance of compression relative to the spring’s resting length), and the constant *x*_0_ being the value of *x* as motion begins. This corresponds to the load placed upon the spring, or the amount of compression of the left ventricle in systole. As the spring is released, the velocity of its recoil is determined by the stiffness constant *k*, where a higher stiffness will lead to a more rapid recoil of the spring, and the energy-loss constant *c*¸ which dampens the recoil. The restoring force at the onset of motion is the product of *k* and *x*_0_, and as the spring moves, loss of energy and thus restoring force is described by the constant *c*. When applied to early diastolic filling, we refer to *c* as the viscoelastic energy loss constant. The E-wave as recorded by PW Doppler describes the filling velocities during the early recoil driven phase of diastole, and therefore describes LV recoil. In the PDF method, the E-wave velocity data is fitted to a mathematical function describing the resulting recoil velocities of any combination of *x*_0_, *k* and *c*; thus each E-wave analyzed yields the constants *x*_0_, *k* and *c*, describing the properties of the LV during that episode of diastolic filling. The PDF method has been extensively validated, [5, 6], and a software program facilitating the application of the method has been described and made freely available [7]. In this study, as a secondary aim, we sought to explore the value of retrospective analysis of a single E-wave per patient.

## Methods

### Study population

Patients undergoing stress echocardiography at the outpatient echocardiography lab at the National Heart Centre Singapore (NHCS) between July 29, 2015 and December 31, 2015 were screened retrospectively for inclusion using data available in an existing clinical echocardiographic database. This population was chosen for the purpose of this study as the stress echocardiography patients at NHCS are rigorously characterized in terms of cardiovascular comorbidities using a dedicated clerking pro-forma which is completed by the attending doctor supervising the test, and all patients undergo 12-lead ECG with electronic documentation made of cardiac rhythm and ECG morphology in each case. Exclusion criteria were resting LV ejection fraction (LVEF) < 55%, more than mild valvular disease, non-sinus rhythm, left bundle branch block, inducible wall motion abnormality (WMA) at stress, and an absence of information on diabetes and hypertension status. Patients with inducible WMA were excluded, since it is established that ischemia affects diastole, and it would be difficult to control for this in the scope of the present study. A subset was extracted for PDF analysis, excluding patients with suboptimal transmitral PW Doppler recordings due to non-evaluable image quality, or fusion of the E- and A-wave involving more than 50% of the maximum velocity of the E-wave.

### Image acquisition

All image acquisition was performed at rest in strict accordance to established chamber quantification guidelines [8] by sonographers trained in echocardiography. The stress protocol was conducted using treadmill exercise following the Bruce protocol in 71% of the subjects, or dobutamine in incrementally increasing doses to a maximum of 40 μg/kg/min in the remaining 29% of subjects. For both protocols, exams not surpassing 85% of maximum predicted heart rate calculates as 220 minus age, were deemed as inconclusive, and not used in the present study. Echocardiography was performed using different scanners in current clinical use. Conventional echocardiographic parameters were recorded by the performing sonographers, and reviewed by an attending cardiologist. E-waves were recorded by placing the pulsed wave (PW) Doppler sample volume at the mitral valve tips as recommended in the guidelines [9]. PDF analysis was performed in a freely available software application, the use of which has been described previously [7]. For the purpose of the analysis of diastolic function, only resting images were available, as the study material consists of examinations performed in a clinical setting, where diastolic parameters are registered only at rest.

### Statistical analysis

Population characteristics are presented as number and frequencies, or medians with interquartile ranges. The effect of the risk factors diabetes, hypertension, sex, and age on the diastolic parameters was investigated using simple and multiple linear regression models, with the diastolic parameters as dependent variables and the risk factors as independent variables. The independent variables were chosen based on clinical knowledge. Normality of residuals and the appropriateness of using linear regression modeling were assessed visually upon inspection of graphical presentation of the results. Results are presented as coefficient estimates with their respective *p*-values, as well as the adjusted R^2^ and overall *p*-values for every model. A *p*-value of < 0.05 was considered statistically significant. Statistical analyses were performed in MATLAB release 2015a (Mathworks, Natick, Massachusetts, USA), and in R 4.0.2 (R Core Team, R Foundation for Statistical Computing, Vienna, Austria).

## Results

**Fig. 1:**
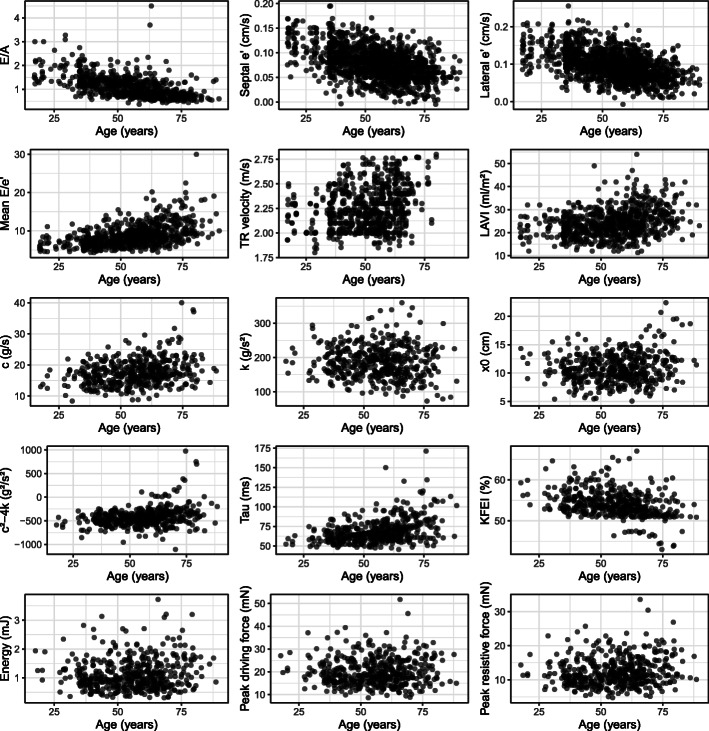
Plots of the diastolic parameters versus age

We included 726 patients, and PDF analysis was performed on a subset of 446 patients. Patient characteristics are described in Table 1, and the same characteristics grouped by presence or absence of diabetes and hypertension are described in Table 2. The results for the conventional diastolic and the PDF parameters are presented in Table 3. The results from the simple linear regression modeling are presented in Table 4. The B coefficients are interpreted as the difference in the mean of the diastolic parameters between patients with and without diabetes, hypertension, male vs female sex, and change per year of age, respectively. For example, patients with hypertension had on average 2.5 cm/s lower lateral e’ velocity compared with patients without hypertension (*p* < 0.001). Similarly, a difference in age from 44 to 65 years (corresponding to the interquartile range of age) was associated with an average decrease of 2.1 cm/s (21 years * -0.1 cm/s/year) in lateral e’ velocity (*p* < 0.001). The results from simple linear regression do not take the effect of combinations of risk factors into account, e.g. the above stated difference in lateral e’ velocity in patients with and without hypertension does not take into account whether these patients also are affected by for example diabetes and high age, but give only the mean difference comparing patients with and without hypertension. In multiple linear regression, these potential combinations of risk factors are explicitly modeled. The results from the multiple linear regression modeling are presented in Table 5. The univariate association between age and the diastolic parameters studied are illustrated in Fig. 1.

**Table 1:** Patient characteristics

	All (***n*** = 726)	PDF subset (***n*** = 446)
Age, years	56 [44–65]	57 [46–65]
Male, n (%)	417 (57)	246 (54)
BMI, kg/m^2^	26 [23–29]	25 [23–29]
BSA, m^2^	1.75 [1.59–1.91]	1.73 [1.57–1.90]
Diabetes, n (%)	311 (43)	235 (52)
Hypertension, n (%)	367 (49)	242 (54)
Heart rate, bpm	75 [67–84]	74 [66–80]
SBP, mmHg	131 [120–143]	130 [121–142]
DBP, mmHg	70 [63–77]	69 [63–76]

Data are shown as median [interquartile range] or n (%). *BMI* Body mass index, *BSA* Body surface area, *SBP* Systolic blood pressure, *DBP* Diastolic blood pressure.

**Table 2:** Patient characteristics by presence or absence of risk factors

	Diabetes	No diabetes	Hypertension	No hypertension
Age, years	61 [63–68]	51 [40–62]	63 [54–70]	49 [38–57]
Male, n (%)	154 (50)	263 (63)	199 (54)	216 (61)
BMI, kg/m^2^	27 [24–30]	24 [22–27]	27 [24–30]	24 [22–27]
BSA, m^2^	1.73 [1.61–1.90]	1.76 [1.58–1-91]	1.74 [1.61–1.91]	1.76 [1.58–1.91]
Heart rate	78 [69–86]	73 [65–81]	76 [68–85]	75 [66–82]
SBP, mmHg	134 [123–146]	128 [119–140]	137 [128–148]	124 [116–135]
DBP, mmHg	70 [63–76]	70 [63–77]	71 [65–77]	68 [62–76]

Data are shown as median [interquartile range] or n (%). *BMI* Body mass index, *BSA* Body surface area, *SBP* Systolic blood pressure, *DBP* Diastolic blood pressure

**Table 3:** Conventional diastolic and PDF parameters

	All (***n*** = 726)	PDF subset (***n*** = 446)
E/A, ratio	1.0 [0.8–1.3]	1.1 [0.8–1.3]
Septal e’, cm/s	7.0 [6.0–9.0]	8.0 [6.0–9.0]
Lateral e’, cm/s	10.0 [8.0–11.0]	10.0 [7.0–11.0]
Mean E/e’, ratio	8.2 [6.8–10.0]	8.6 [7.1–10.6]
Mean E/e’ > 14	31 (4.3)	25 (5.6)
TR velocity, m/s	2.4 [2.2–2.6]*	2.2 [2.4–2.5]
LAVI, ml/m^2^	24 [20–28]	25 [21–28]
Diastolic function grade		
Normal	675 (93.0)	409 (91.2)
Grade I dysfunction	2 (0.3)	1 (0.2)
Grade II dysfunction	9 (1.2)	7 (1.6)
Grade III dysfunction	1 (0.1)	1 (0.2)
Indeterminate	39 (5.3)	28 (6.2)
*c*¸ g/s		17.1 [14.6–19.7]
*k*, g/s^2^		185.7 [154.7–216.9]
*x*_0_, cm		10.6 [9.2–12.5]
*c*^2^-4*k*, g^2^/s^2^		-439.1 [- 536.0− -343.8]
Tau, ms		65.5 [58.9–74.8]
KFEI, %		53.6 [52.1–55.8]
Energy, mJ		1.0 [0.7–1.4]
Peak driving force, mN		18.8 [15.8–23.9]
Peak resistivs force, mN		11.5 [9.4–14.8]

Data are shown as median [interquartile range] or n (%). *TR* Tricuspid regurgitation, *LAVI* Left atrial volume index, *PDF* The parameterized diastolic filling method, *c* viscoelastic energy loss, *k* Stiffness, *x*_*0*_ Load, *KFEI* Kinematic filling efficiency index. *) The highest recorded velocity in any patient was 2.8 m/s

**Table 4:** Simple linear regression models

	Diabetes			Hypertension			Sex (male)			Age (per year)		
	B	R^**2**^	p	B	R^**2**^	p	B	R^**2**^	p	B	R^**2**^	p
LAVI, ml/m^2^	**1.0**	0.01	**0.036**	**2.5**	0.04	**< 0.001**	**−1.7**	0.02	**< 0.001**	**0.12**	0.09	**< 0.001**
Septal e’, cm/s	**−1.8**	0.15	**< 0.001**	**−2.1**	0.21	**< 0.001**	**0.5**	0.01	**0.002**	**−0.1**	0.42	**< 0.001**
Lateral e’, cm/s	**−2.1**	0.12	**< 0.001**	**−2.6**	0.18	**< 0.001**	**1.1**	0.03	**< 0.001**	**−0.13**	0.40	**< 0.001**
E/A, ratio	**−0.3**	0.08	**< 0.001**	**−0.3**	0.14	**< 0.001**	**0.1**	0.01	**0.004**	**− 0.02**	0.32	**< 0.001**
Mean E/e’, ratio	**1.9**	0.10	**< 0.001**	**2.2**	0.14	**< 0.001**	**−1.5**	0.07	**< 0.001**	**0.1**	0.24	**< 0.001**
*c*¸ g/s	**1.0**	0.01	**0.014**	**1.6**	0.03	**< 0.001**	**−1.6**	0.04	**< 0.001**	**0.09**	0.08	**< 0.001**
*k*, g/s^2^	**−12.5**	0.02	**0.005**	−7.6	< 0.01	0.09	**−18**	0.04	**< 0.001**	−0.21	< 0.01	0.20
*x*_0_, cm	**0.8**	0.02	**0.002**	**0.8**	0.02	**0.001**	**−0.5**	0.01	**0.028**	**0.03**	0.02	**0.003**
*c*^2^-4*k*, g^2^/s^2^	**91**	0.05	**< 0.001**	**93**	0.05	**< 0.001**	12.5	< 0.01	0.51	**4.4**	0.09	**< 0.001**
Tau, ms	**6.3**	0.04	**< 0.001**	**8.3**	0.07	**< 0.001**	−1.6	< 0.01	0.26	**0.45**	0.16	**< 0.001**
KFEI, %	**−1.3**	0.04	**< 0.001**	**−1.7**	0.06	**< 0.001**	**0.7**	0.01	**0.016**	**−0.09**	0.13	**< 0.001**
Energy, mJ	0.1	< 0.01	0.19	0.1	0.01	0.05	**−0.2**	0.04	**< 0.001**	0.003	< 0.01	0.07
Peak driving force, mN	−0.1	< 0.01	0.80	0.3	< 0.01	0.55	**−3.0**	0.06	**< 0.001**	0.01	< 0.01	0.58
Peak resistive force, mN	0.4	< 0.01	0.35	0.8	0.01	0.05	**−2.1**	0.06	**< 0.001**	**0.04**	0.01	**0.009**

*LAVI* Left atrial volume index.. *c* Viscoelastic energy loss, *k* Stiffness, *x*_0_ Load, *KFEI* Kinematic filling efficiency index. R^2^ is adjusted R^2^

**Table 5:** Multiple linear regression models

Dependent	Diabetes		Hypertension		Sex (male)		Age (per year)			
	B	p	B	p	B	p	B	p	R^**2**^	p*
LAVI, ml/m^2^	− 0.7	0.16	**1.4**	**0.007**	**− 0.9**	**0.04**	**0.10**	**< 0.001**	0.10	< 0.001
Septal e’, cm/s	**−0.8**	**< 0.001**	**− 0.7**	**< 0.001**	− 0.2	0.09	**− 0.09**	**< 0.001**	0.48	< 0.001
Lateral e’, cm/s	**−0.8**	**< 0.001**	**− 0.8**	**< 0.001**	0.2	0.21	**−0.10**	**< 0.001**	0.44	< 0.001
E/A, ratio	**−0.10**	**0.002**	**−0.10**	**0.004**	−0.03	0.34	**−0.02**	**< 0.001**	0.34	< 0.001
Mean E/e’, ratio	**0.8**	**< 0.001**	**0.9**	**< 0.001**	**− 0.9**	**< 0.001**	**0.07**	**< 0.001**	0.31	< 0.001
*c*¸ g/s	0.01	0.98	0.7	0.10	**−1.3**	**0.001**	**0.07**	**< 0.001**	0.10	< 0.001
*k*, g/s^2^	**−12**	**0.01**	−0.4	0.94	**−20**	**< 0.001**	− 0.20	0.29	0.05	< 0.001
*x*_0_, cm	0.4	0.10	0.4	0.12	−0.4	0.07	0.01	0.22	0.03	0.001
*c*^2^-4*k*, g^2^/s^2^	**52**	**0.009**	30	0.16	33	0.07	**3.6**	**< 0.001**	0.11	< 0.001
Tau, ms	2.0	0.19	3.0	0.06	0.3	0.82	**0.39**	**< 0.001**	0.17	< 0.001
KFEI, %	−0.4	0.18	**−0.7**	**0.04**	0.4	0.18	**− 0.068**	**< 0.001**	0.14	< 0.001
Energy, mJ	0.01	0.85	0.09	0.14	**−0.22**	**< 0.001**	0.0005	0.83	0.04	< 0.001
Peak driving force, mN	−0.6	0.36	0.7	0.29	**−3.1**	**< 0.001**	−0.01	0.59	0.06	< 0.001
Peak resistive force, mN	−0.2	0.66	0.6	0.17	**−2.1**	**< 0.001**	0.02	0.29	0.07	< 0.001

*LAVI* Left atrial volume index. *c* Viscoelastic energy loss, *k* Stiffness, *x*_0_ Load, *KFEI* Kinematic filling efficiency index. R^2^ is adjusted R^2^. *These *p*-values are for the overall model using the F-test

## Discussion

The present study describes the association between diabetes, hypertension, sex, age, and various parameters of diastolic function. The cohort of patients used all had an LVEF > 55%, and absence of signs of ischemic heart disease on stress echocardiography, the latter to reduce the risk of various degrees of ischemia resulting in acute changes in diastolic function. As expected, diabetes and hypertension and increasing age were associated with lower tissue Doppler velocities, lower E/A-ratio, higher E/e’ and higher LAVI. In the PDF framework, these risk factors were associated with higher viscoelastic energy loss and lower stiffness, which, by definition, corresponds to a lower tendency of the LV to recoil during early diastole. Notably, when using multiple linear regression to study the effect of the coexistence of these risk factors, the negative impact of diabetes and hypertension was attenuated, with increasing age retaining much of its negative effect. Furthermore, age explained a substantially larger fraction of the variance in conventional diastolic parameters compared with diabetes, hypertension, and sex, and adding the other risk factors to age in a multiple regression model only slightly increased this fraction.

For the PDF parameters, the studied risk factors did not explain any substantial part of the variance, and, notably, age only exerted a minor effect on these parameters. These findings are somewhat counterintuitive, and could likely be explained in part by an insufficient number of E-waves analyzed per subject. Since the PDF method uses the E-wave as its only data input, it is possible that, compared to other paradigms using several related measurements (e.g. E/e’), the importance of averaging over several E-waves to decrease noise becomes important. However, the relatively large number of subjects studied likely attenuates in part the lack of averaging of multiple measures per subject.

Comparing our findings to some of the previous research in this area, an increased prevalence of mild, but not severe, diastolic dysfunction has been found among patients with hypertension or diabetes, compared to healthy controls [10]. That study also found similar sizes of the regression coefficients for diabetes and hypertension [10]. Substantially smaller differences for age- and sex adjusted e’ and E/e’ have been found when comparing hypertensive patients with or without left ventricular hypertrophy with healthy controls [11]. Also, similar results have been found regarding the differences in conventional diastolic parameters comparing diabetic and non-diabetic patients [12]. However, by comparison, the present study emphasizes that increasing age seems to be a factor with a sizable association with diastolic dysfunction, even in the setting of concurrent diabetes or hypertension.

### Limitations

The studied population represents a convenience sample of patients undergoing investigation with stress echocardiography. The population is well characterized in terms of medical history and current findings from echocardiography, and results from stress echocardiography. Furthermore, the retrospective nature of patient identification may introduce confounding factors. However, given the size of the dataset it is likely to reflect a real-life clinical situation. A further limitation is the classification of patients as having or not having diabetes and hypertension. It seems likely that the duration of these conditions in a given patient would be of importance for the development of diastolic dysfunction, but we did not have access to this information. We also did not have access to data on patient medication, which may have influence on diastolic parameters. Furthermore, we actively excluded patients with stress echocardiography findings of ischemic heart disease. It is possible that this may have attenuated the effect of diabetes and hypertension on diastolic function, since those excluded are more likely than others to be diabetic with poor disease control. However, given the retrospective nature of this study, our assessment was that the group with pathological stress echocardiography findings would likely be heterogeneous and precarious to interpret. For the PDF analysis, we were limited to analyzing one E-wave per patient. In our opinion, to optimally apply this method, several E-waves should be analyzed for every subject, due to the noisy nature of Doppler recordings and variable effect of breathing and other loading conditions on mitral inflow.

### Conclusions

In conclusion, diabetes, hypertension, increasing age, and to a lesser extent sex, explain a moderate amount of the variance in conventional diastolic parameters in a healthy outpatient population. The effect of these risk factors was substantially less pronounced on left atrial volume index and the PDF parameters. Age seems to be the most important risk factor.

## Data Availability

The data that support the findings of this study are available from NHCS but restrictions apply to the availability of these data, which were used under a specific agreement for the current study, and so are not publicly available. Data are however available from the authors upon reasonable request and with permission of NHCS.

## Abbreviations

HFpEF: Heart failure with preserved ejection fraction
LVEF: Left ventricular ejection fraction
NHCS: National Heart Centre Singapore
PDF: Parameterized diastolic filling
PW Doppler: Pulsed wave Doppler
